# Towards an integrated view of vocal development

**DOI:** 10.1371/journal.pbio.2005544

**Published:** 2018-03-22

**Authors:** Gabriel B. Mindlin

**Affiliations:** 1 Departamento de Física, FCEyN, Universidad de Buenos Aires, Bueno Aires, Argentina; 2 IFIBA, CONICET, Buenos Aires, Argentina

## Abstract

Vocal development is usually studied from the perspective of neuroscience. In this issue, Zhang and Ghazanfar propose a way in which body growth might condition the process. They study the vocalizations of marmoset infants with a wide range of techniques that include computational models and experiments that mimic growth reversal. Their results suggest that the qualitative changes that occur during development are rooted in the nonlinear interaction between the nervous system and the biomechanics involved in respiration. This work illustrates how an integrative approach enriches our understanding of behavior.

## Integrated views

Behavior emerges from the interaction between nervous system, body, and environment [1]. Not many researchers would disagree with this statement, and yet when it comes to addressing a behavioral problem, this integrative view is often left aside. There are exceptional cases in which all these elements have been incorporated into the analysis of the problem. For example, studies have shown that the beautiful swimming pattern of the lamprey emerges through the interaction of specifically connected Central Pattern Generators (CPGs), particular biomechanics, and the interaction of the animal’s body with the aquatic medium [2]. Even though it is not surprising that the study of a locomotion problem incorporates a biomechanical perspective, other behavioral problems seem to be almost exclusively analyzed with a disregard for the biomechanics involved.

One such problem is vocal development. Typically, the studies in this field focus on how the changes in the neural circuitry involved affect the vocal output. The imitative aspect of vocal learning has been studied both at the level of the nervous system (particularly in humans and in songbirds [3], with almost a complete absence of data for nonhuman mammalian species), as well as at the level of the social interaction required [4]. Memory, perceptual predispositions, and auditory–motor mappings have been studied in depth, but the role played by the changes in the body structure that generates this behavior has been much less studied. Is it possible that the changes that occur in the biomechanics during development affect qualitatively some aspects of vocal development? This is the question that Zhang and Ghazanfar address in “Vocal development through morphological computation” [5], a study of the vocalizations produced by marmoset monkeys in their first two months of life, in what constitutes the first approach to investigate this topic in a nonhuman mammalian species. In particular, the authors were able to interpret the progressive loss of specific calls and the elongation of others during development as a consequence of lung growth, without the need to invoke changes at the neural level. Let us see which are the tools that are needed to carry out a study that is both conceptually integrative and specific in its predictions.

## Nonlinear dynamics

To predict something as specific as the families of pressure patterns used for birdsong production [6], the spatiotemporal symmetries of the quadrupedal gaits [7], or the precise motor patterns required to produce the vocalizations of a marmoset monkey [8], we have to move beyond conceptual models and work with computational ones. Of course, it is not always easy, particularly when behavior is involved. One major problem with this approach is that, unlike physics—which has built a solid bridge between the physics of one particle and the macroscopic world (that bridge is called statistical mechanics)—there are no (finished) bridges linking our understanding of the behavior of one neuron to the parameters controlling a macroscopic biomechanical device. Therefore, we have to rely on phenomenology and educated intuition in order to identify pertinent macroscopic variables for our problem. For example, simple respiratory models have been written in terms of variables describing the level of activity of two mutually inhibiting neural populations and a variable describing the lung volume [9].

What has been imported from physics is the idea that, if we are interested in the fate of some variables, we need to model how their temporal rates of change depend on all the variables of the problem. The reason for doing this is that, if we know the value of the variables at one instant, we can predict their value after a small amount of time by adding to their present values the rates of change multiplied by the small time increment. That is the reason behind computational models being written in terms of differential equations. Dynamics is precisely the branch of mathematics that uses information about the state of a system in order to predict its temporal evolution. When the rules that prescribe those rates of change are nonlinear functions of the variables, we speak of nonlinear dynamics [10,11].

There has been enormous progress in nonlinear dynamics in the last few decades, mostly in the development of tools that allow us to obtain qualitative information on the expected dynamics of a system, without the need to calculate an analytical solution. One of these tools is the bifurcation diagram. It is a plot with axes representing the parameters of the system (i.e., the numbers that describe the system’s configuration). In this plot, one displays curves that indicate boundaries between regions of parameter space. Within each region, the variables of the problem behave in a qualitatively similar way.

## Biophysics of phonation

Production of human voiced sounds [12], birdsong [13,14], and marmoset calls [8] share some important features. All of them consist of some valve, set in motion as subglottal pressure exceeds a threshold. Therefore, there are two timescales involved: the rapid valve oscillation (responsible for the pitch) and the slow subglottal variation (responsible for the rhythmicity of the vocalization). During the phonation, other parameters that control the frequency of the oscillations can present a slow variation as well. In birdsong production, it is the activity of the muscles controlling the configuration of the syrinx. Humans exhibit a stellar display of motor gestures that affect the sound filtering at the timescale of the phoneme. In the vocalization of the marmoset, it is the laryngeal tension.

**Fig 1:**
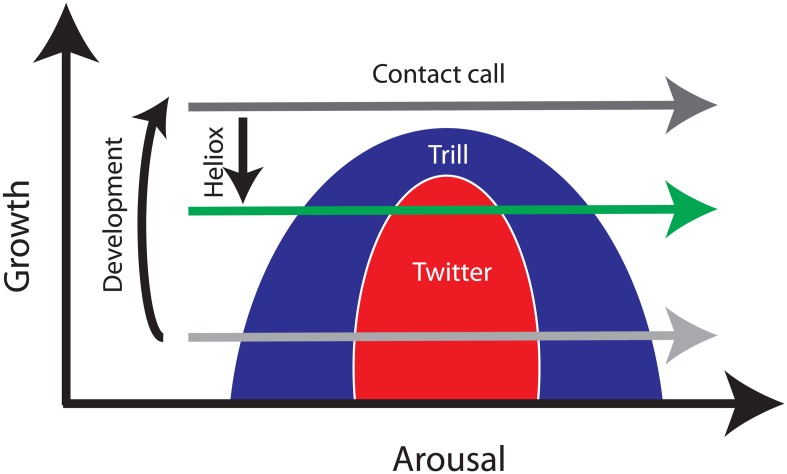
The schematics of a bifurcation diagram and its use in experimental design. A computational model for slow motor gestures predicts the existence of three regions of the parameter space [5]. For parameters in each region, qualitatively different solutions (different behaviors) are expected. One of the parameters is related to the animal’s growth. As the second parameter is varied, different solutions can be found at early stages of development (light grey arrow), and only one solution type is expected later (dark grey arrow). Placing marmoset infants in a heliox atmosphere, Zhang and Ghazanfar mimic the reversal of a parameter that correlates with development, recovering the lost behaviors (green arrow).

The model built and discussed by Zhang and Ghazanfar [5] involves the slow gestures (subglottal pressure and laryngeal tension). Studying their model, one notices that they identify three qualitatively different regimes. In the first one, the pressure fluctuates slowly, while the tension is constant. In the second one, the tension starts to oscillate at a somewhat higher rate, inducing small fluctuations on top of the slow subglottal fluctuations. In the third regime, there are large fluctuations in both laryngeal tension and subglottal pressure. One interesting thing about their model is that, when they feed these simulated gestures into a phonating model, they can synthesize the different marmoset vocalizations. Yet another important result is that it is possible to build a bifurcation diagram in which one of the axes is proportional to the inertia opposed by the lungs. Because that parameter is expected to increase its value during the growth of the animal, it is possible to explore how the regions of the parameter space with different solution types change as the “growth” parameter is varied (see Fig 1). In this way, it is possible to predict precisely which solutions are expected to gradually disappear during development. This mechanism successfully accounts for the decreasing proportion of two families of vocalizations as the feedback from the lungs varies consistently with body growth. Furthermore, it is possible to predict the outcome of an experiment in which the parameters of the model are manipulated. To that effect, Zhang and Ghazanfar placed infant marmosets in a helium–oxygen atmosphere (a lighter gas than the normal atmosphere), emulating mechanically a reversal of the body growth. Consistent with the predictions of their model, the infant marmosets recovered the vocalizations that had been lost during development.

## Discussion

In the study of locomotion, in which neural circuits generate patterns that are coupled to the environment by the body–limb system, the importance of the biomechanics involved is clear [2]. In other problems of behavior, this integrative view has not been fully embraced yet. The loss of well-coordinated stepping behavior in human infants after the age of two months was shown to be due to body growth (and not to neural changes) by Thelen and colleagues in the 1980s [15]. In a similar spirit, Zhang and Ghazanfar show now that marmoset monkeys undergo changes in their vocalizations, which can be explained in terms of how the nervous system and the body interact [5]. These two temporally distant examples show that, even in problems that would be naturally explored from the perspective of pure neuroscience, an integrative view enriches our understanding of development. In the field of vocal production, the work by Zhang and Ghazanfar can be framed within a small set of studies that highlight the interplay between neuronal activity and the dynamics of the vocal organ to explain vocal structures [6,16,17].

The integrative perspective poses challenges: the larger the number of subsystems, the larger the number of observables to follow. And because the interactions between them will typically be nonlinear, predicting their outcome under some hypothetical interaction is bound to be complicated. It is precisely for this reason that computational models can help in the interpretation of existing data as well as in the design of new behavioral experiments. Nonlinear models, and specifically the concept of bifurcation, might be a natural language to study the qualitative changes that so often characterize developmental changes. Granted, the phenomenological nature of the models could be a problem when it comes to interpreting negative results because it is not possible to know whether it is our specific hypothesis or the basic model that is being refuted. However, the confidence provided by the positive results that emerge from the dialogue between quantitative models and the experiments are worth the effort. In that regard, the work of Zhang and Ghazanfar constitutes an outstanding example.

## Abbreviation

CPG: Central Pattern Generator
